# Supplementary material for: Extracellular electron transfer-dependent anaerobic oxidation of ammonium by anammox bacteria
Source: Nat Commun. 2020 Apr 28;11:2058. doi: 10.1038/s41467-020-16016-y (PMC7188810; doi:10.1038/s41467-020-16016-y)
Supplement: Supplementary file 1 — Supplementary Information [file 41467_2020_16016_MOESM1_ESM.pdf]

Supplementary information for

Extracellular electron transfer-dependent anaerobic oxidation of ammonium by anammox bacteria

By Shaw et al.

Supplementary discussion

Putative EET-dependent anammox pathway

We provided evidence that phylogenetically distant anammox bacteria can perform EET and are electrochemically active, and we elucidated the molecular mechanism of NH_4^+ oxidation, which by itself are significant findings that changes our perception of a key player in the global nitrogen cycle. Next, we conducted a comparative transcriptomic analysis to identify the possible pathways involved in the EET-dependent anammox process (electrode poised at 0.6 V vs. standard hydrogen electrode (SHE) the as electron acceptor) versus typical anammox process (i.e., NO_2^- as the electron acceptor). Currently, pure cultures of anammox bacteria are unavailable to conduct mutant studies to address the genetic basis of EET-dependent anammox process[1]. Also, the slow growth rates of anammox bacteria and the fact that they do not rapidly degrade the majority of their proteins, make the changes to specific conditions (i.e., changes in the electron acceptor) not immediately reflected at the protein level[2]. Therefore, to detect immediate changes in response to a stimulus, short-term gene expression responses would be more appropriate for this aim. In our study, the potential metabolic pathways involved in EET-dependent anammox process, were studied using a genome-centric stimulus-induced transcriptomics approach that has been successfully applied before to identify metabolic networks within complex EET-active microbial communities[3]. RNA samples were extracted from mature electrode biofilm of two independent single-chamber *Ca*. Brocadia MECs operated first with NO_2^- as the sole electron acceptor and after switching to set potential growth (0.6 V vs. SHE), and were subjected to a comparative transcriptomics analysis. Similar experiments were conducted with *Ca*. Scalindua and *K. stuttgartiensis*, but we did not get sufficient mRNA from the biofilm samples, and hence only the

data for *Ca*. Brocadia are presented here. High similarity was observed between the biological replicates and differentially expressed genes across the experimental setups (Supplementary Fig. 9). Based on the known cell biology[4], biochemistry and anammox metabolism[5–7], and the expression profiles of the known anammox pathways obtained with the differential expression analysis done in this study (Supplementary Tables 3 and 4), we propose a putative molecular model to describe how electrons flow from the anammoxosome to the electrode in the EET-dependent anammox process (Supplementary Fig. 10). The most differentially expressed genes in response to the change to the electrode as electron acceptor were mainly associated with energy conservation and nitrogen metabolism (Supplementary Tables 6 and 8). The metabolic challenge that must be solved for EET process in anammox cells is to transfer the electrons through the separate compartments and membranes (anammoxosome, cytoplasm and periplasm). The observed NH_4^+ oxidation and reproducible current generation can only be explained by electrons being transported from the anammoxosome (energetic central of the cell and where the NH_4^+ is oxidized) to the electrode. In the anammoxosome, the genes encoding for ammonium transporters (AmtB), a hydroxylamine oxidoreductase (HAO) and hydrazine dehydrogenase (HDH) were the most upregulated (Supplementary Fig. 10, Supplementary Table 8). This result is consistent with the NH_4^+ uptake, oxidation and final conversion to N_2 observed in the MECs and isotope labeling experiments (Fig. 2a, Fig 3a). The requirement of more moles of NH_4^+ when anammox growth is based on EET compared to NO_2^- as electron acceptor (Eq. 1), increases the demand of NH_4^+ import into the cell, which can explain the upregulation of the ammonium transporters. In contrast, the genes encoding for NO and NO_2^- reductases (*nir* genes) and their redox couples were significantly downregulated (Supplementary Fig. 10, Supplementary Table 8). This agrees, with the fact that NO_2^- and NO_3^- were below the detection limit in the MECs (Fig. 2a, Supplementary Fig. 3a and

b) and there was no effect of PTIO when NO_2^- was replaced by electrode as the electron acceptor (Supplementary Fig. 8). Also, this supports the hypothesis that NO is not an intermediate of the electrode-dependent anammox process. The most downregulated HAO in the electrode-dependent anammox process (EX330_09385, Supplementary Table 8) is an ortholog of the proposed nitrite reductase in *K. stuttgartiensis* kustc0458[7]. Currently, nitrite reductase(s) responsible for NO_2^- reduction in *Brocadia* species are unidentified[8]. Therefore, it would be of interest to further investigate the function of the downregulated HAO found in this study as possible candidate for *nir* in *Brocadia*. On the other hand, the *nxr* genes encoding for the soluble nitrite:nitrate oxidoreductase maintained similar levels of expression under both conditions (Supplementary Table 9). However, cytochromes of the *nxr* gene cluster and the hypothetical membrane-bound NXR were found downregulated under set-potential (Supplementary Table 8). Even though ammonia is difficult to activate under anaerobic conditions[9], previous studies have reported anaerobic NH_4^+ oxidation in bioelectrochemical systems dominated by nitrifiers[10–15], but the molecular mechanism was not elucidated. Also, an alternative process to anammox called Feammox has been reported recently, where NH_4^+ oxidation is coupled with Fe(III) reduction by the Actinobacteria *Acidimicrobiaceae* sp. A6[16,17]. It should be noted that *Acidimicrobiaceae* sp. A6 is not recognized as a key player in the nitrogen cycle. When a pure culture of *Acidimicrobiaceae* sp. A6 was tested in MECs with an electrode as electron acceptor, there was no colonization and biofilm formation over the course of the experiment. The majority of *Acidimicrobiaceae* cells were present in suspension in the MECs, which explains the low Coulombic Efficiency of the process (~16.4%) and the need for the soluble electron shuttle 9,10-anthraquinone-2,6-disulfonic acid (AQDS). In the absence of AQDS, no change in $NH4^+$ concentration was detected. Future experiments are needed to differentiate Fe(III) reduction for

nutritional acquisition from respiration through EET, and to address the genetic basis of the Feammox process and elucidate the molecular mechanism of NH_4^+ oxidation. Our isotope labelling experiments revealed that NH_2OH is a key intermediate in the oxidation of NH_4^+ in electrode-dependent anammox process (Fig. 3b), suggesting that the internalized NH_4^+ is oxidized to NH_2OH. More than 10 paralogs of HAO-like proteins in anammox are the most likely candidate enzymes catalyzing anaerobic NH_4^+ oxidation. The only upregulated HAO-like protein (EX330_11045) (Supplementary Fig. 10, Supplementary Table 8), whose function is still uncharacterized, lacks the tyrosine residue needed for crosslinking of catalytic heme 4, thereby favoring reductive reactions[8]. This HAO is an ortholog of *K. stuttgartiensis* kustd2021 which under normal anammox conditions has low expression levels[18]. However, it is worth mentioning that under set potential the whole gene cluster EX330_11030-11050 was significantly upregulated (Supplementary Table 10). Thus, further investigation should focus on determining the role of this cluster in electrode-dependent anammox process. The produced NH_2OH is then condensed with NH_3 to produce N_2H_4 by the hydrazine synthase (HZS)[19] (Supplementary Fig. 10). Recent crystallography study of *Ca. K. stuttgartiensis* HZS, suggested that N_2H_4 synthesis is a two-step reaction: NO reduction to NH_2OH and subsequent condensation of NH_2OH and NH_3[19]. Our isotope labelling experiments showed that NH_2OH is an intermediate in the electrode-dependent anammox process, and thus there is no need for the reduction of NO to NH_2OH, which explains the downregulation of the electron transfer module (ETM) and its redox partner (Supplementary Fig. 10, Supplementary Table 6). Under "normal" anammox conditions (i.e., NO_2^- as electron acceptor), the membrane associated quinol-interacting ETM encoded in the HZS gene cluster, mediate the first half-reaction for N_2H_4 synthesis[7,20]. The ETM provides three-electrons to the HZS enzymatic complex for NO reduction to NH_2OH with the help of an electron shuttle[20,21]. N_2H_4 is

further oxidized to N_2 by HDH (Supplementary Fig. 10). The four low-potential electrons released from this reaction must be stored until they are transferred to a redox partner and feed the quinone (quinol) pool within the anammoxosome membrane to build up the membrane potential[22]. Currently, it is not known how electrons are transported over membranes when NO_2^- is the electron acceptor. Understanding the electron flow and electron carriers is an important next step in anammox research. A recent exciting study showing the structure of the HDH[22], revealed that HDH can store up to 192 electrons and it is proposed that the appropriate carriers might specifically dock into the enzyme to get the electrons and transport them to the desired acceptor. This will prevent accidental transfer of the low-redox potential electrons to random acceptors. Interestingly, HDH was one of the most upregulated enzymes in our study when the anode was the electron acceptor, which suggests an increased demand of electron storage and transport when the anode is the electron acceptor. In the typical anammox process, quinol oxidation supplies electrons for the reductive steps, thus closing the electron transfer cycle. However, under electrode-dependent anammox process, where there is no NO_2^- and the electrode is the sole electron acceptor, electrons must first pass to the cytoplasm. By accepting the electrons from N_2H_4 oxidation, energy would be conserved as reduced quinone and NAD(P)H, which can work as electron carrier in the cytoplasm. This set of reactions are thermodynamically feasible and are done by the Rieske/cytb complexes of anammox bacteria[7] (See 'Respiratory complexes of anammox bacteria in EET-dependent anammox process' section).

Several genes encoding for low-molecular-weight mobile carriers dissolved in the cytoplasm (NADH, ferredoxins, rubredoxins) were found expressed under set potential conditions (Supplementary Fig. 10, Supplementary Table 3). These low-molecular-weight electron carriers act as electron shuttles between the respiratory complexes in the anammoxosome and the central

carbon and iron metabolism of anammox bacteria[6,7]. Even though non-heme electron carriers dissolved in the cytoplasm are proposed as electron shuttles, it is still not clear how the electrons are transferred from the respiratory complexes in the anammoxosome to the inner membrane, even when NO_2^- is the electron acceptor. Our transcriptomic data identified an EET pathway in *Ca*. Brocadia in response to the electrode as the electron acceptor. The EET pathway found in anammox bacteria is analog to the ones present in metal-reducing organisms such as *Geobacter* spp. and *Shewanella* spp[23]. To overcome the membrane barriers, electrons from the oxidation of menaquinol by an inner-membrane tetraheme *c*-type cytochrome (Cyt *c* (4 hemes)) are transferred to the periplasmic mono-heme *c*-type cytochrome (Cyt *c* (1 heme)) (Supplementary Fig. 10, Supplementary Table 3). The tetraheme *c*-type cytochrome (Cyt *c* (4 hemes)), may function as a quinol dehydrogenase of the EET cascade, similar to the role played by the tetraheme CymA in *Shewanella*[24,25]. The highly upregulated mono-heme cytochrome c (Cyt *c* (1 heme)) was found to have homology with MtoD of the metal-oxidizing bacteria *Sideroxydans lithotrophicus* ES-1[26]. MtoD has been characterized as a periplasmic monoheme cytochrome *c* that works as an electron shuttle between CymA and outer membrane cytochromes[26]. It is still not clear which protein(s) feed the menaquinol pool used by the tetraheme *c*-type cytochrome in the inner membrane. It has been reported that for EET in *S. oneidensis*, electrons could enter the inner-membrane pool via the activity of primary dehydrogenases, such as NADH dehydrogenases, hydrogenases or formate dehydrogenase (Fdh)[27]. Also, a previous study revealed formate oxidation coupled with Fe(III) or Mn(IV) reduction in anammox bacteria[28]. In our analysis, we found a significant expression under set potential of multiple copies of the Fdh and its transcriptional activator (Supplementary Fig. 10, Supplementary Table 3), which possibly are involved in the EET pathway to respire insoluble minerals in anammox bacteria. It has been reported that C1 metabolism such as formate oxidation

by Fdh is strongly related to the electron transferring to the extracellular environment[27]. Evidence suggests that formate can act as a stimulus for external electron transfer in the absence of soluble electron acceptors, which is related to the existence of a periplasmic Fdh to convert formate to CO_2 with the electrons being released extracellularly[27]. Similar to *S. oneidensis*, *Ca*. Brocadia electricigens gets a significant amount of proton motive force and feeds the quinol pool in the inner membrane by transporting and oxidizing formate in the periplasm[29] (Supplementary Fig. 10).

Outer membrane protein complexes can transfer the electrons from the periplasm to the bacterial surface via an electron transport chain[24]. The wide windows of these cytochromes allow an overlapping of redox potentials in an electron transport chain and make possible a thermodynamic downhill process of electron transport[30]. It has been reported that *K. stuttgartiensis* possesses a trans-outer membrane porin-cytochrome complex for extracellular electron transfer that is widespread in different phyla[31,32]. The genes encoding for the porin-cytochromes are adjacent to each other in the genome (kuste4024 and kuste4025) and consist of a periplasmic and a porin-like *c*-type outer-membrane cytochrome[31,32]. As expected, *Ca*. Brocadia electricigens expressed the ortholog of the outer-membrane porin-cytochrome complex (Supplementary Fig. 10, Supplementary Table 3). Compared to the porin-cytochrome complexes of six different phyla, anammox bacteria porin-cytochromes are larger and possess more heme-binding motifs[32]. This may provide anammox bacteria a sufficient span to transfer electrons across the outer membrane without the need of additional outer-membrane cytochromes[32]. However, biofilm CV analysis (Fig. 2e, Supplementary Fig. 3c and d) exhibited oxidation/reduction peaks, which suggests that additional cytochrome(s) that transfer electrons directly to the electrode via solvent exposed hemes may be involved. Also, no cytochromes for long-range electron transport were detected in the analysis (Supplementary Table 6 and 7), suggesting that EET to electrodes by anammox bacteria

rely on a direct EET mechanism. Homology detection and structure prediction by hidden Markov model comparison (HMM-HMM) of the highly upregulated penta-heme cytochrome EX330_07910 (Supplementary Fig. 10, Supplementary Table 6) gave high probability hits to proteins associated to the extracellular matrix and outer membrane iron respiratory proteins such as MtrF, OmcA and MtrC. Also, it is worth mentioning that the gene cluster EX330_07910-07915 was one of the most upregulated under set-potential conditions. Therefore, future work should focus on determining the role of EX330_07910- 07915 in the EET-dependent anammox process. Likewise, we also found the expression of outer membrane mono-heme *c*-type cytochromes (OM Cyt c (1 heme) (Supplementary Fig. 10, Supplementary Table 7) homologs to *G. sulfurreducens'* OmcF, which has been characterized to be an outer membrane-associated monoheme cytochrome involved in the regulation of extracellular reduction of metal oxides[33]. Anammox bacteria have a diverse repertoire of conductive and electron-carrier molecules that can be involved in the electron transfer to insoluble electron acceptors. Therefore, it is possible that different pathways may be involved in parallel in the EET-dependent anaerobic ammonium oxidation

Respiratory complexes of anammox bacteria in EET-dependent anammox process

In the current proposed model of the anammox process, the four electrons released from the N_2H_4 oxidation are transferred to the menaquinone pool in the anammoxosome membrane by the action of a yet unknown oxidoreductase[7,22]. The resulting proton gradient across the anammoxosome membrane drive the adenosine 5′-triphosphate (ATP) synthesis[22]. However, in general, little is known about how anammox bacteria transport and utilize the energy released by the N_2H_4 oxidation in the respiratory complexes in the anammoxosome membrane[22]. These respiratory processes depend heavily on membrane-bound complexes such as the *bc1* complex[6]. It is proposed

that the Rieske/cytb *bc1* complex in anammox bacteria plays a central role coupling the oxidation of two-electron carrier quinol with the reduction of two *c*-type cytochromes with a net proton translocation stoichiometry of $4H^+/2e^-$[6]. With this electron bifurcation mechanism, it is thermodynamically feasible to synthesize NAD(P)H by coupling oxidation of (mena)quinol to the reduction of an electron acceptor of higher redox potential such as NAD(P)[6]. A previous study revealed that in the typical anammox process (i.e., NO_2^- as electron acceptor), gene products of Rieske/cytb *bc1* and *bc3* of anammox bacteria were the least and most abundant complexes in the anammoxosome membrane, respectively[7]. In contrast, our comparative transcriptomics analysis revealed that with an electrode as the sole electron acceptor, complex *bc1* and *bc3* were upregulated and downregulated, respectively (Supplementary Fig. 10, Supplementary Table 6). In agreement with the current knowledge of anammox biochemistry[6,7], in our model, we also propose a bifurcation mechanism for NAD(P)H generation in concert with menaquinol oxidation catalyzed by the *bc1* complex and/or a H^+ translocating NADH:quinone oxidoreductase (NADH dehydrogenase, NADH-DH). The energy released by NADH oxidation to quinone reduction ($\Delta G^{0'}$ = -47 kJ) can be utilized by the upregulated sodium-dependent NADH:ubiquinone oxidoreductase (RnfABCDGE type electron transport complex) to translocate sodium ions, thus creating a Na-motive force[6] (Supplementary Fig. 10). Accordingly, a Na-motive force might be employed to drive the opposite unfavorable NAD^+ reduction by the upregulated NAD-dependent oxidoreductases, quinol dehydrogenases or NAD-dependent dehydrogenase[6] (Supplementary Fig. 10, Supplementary Table 3). In the membrane-bound Rnf complex, the electrons from the oxidation of NADH are transferred to ferredoxins (Fd_{red})[7]. Since redox potential of Fd ($E^{0'}_{Fd}$ = -500 to -420 mV) is more negative than NAD+/NADH couple ($E^{0'}_{NADH}$ = -320 mV), the excess energy is available for transmembrane ion transport[30]. Ferredoxins act as non-heme electron

carriers in the cytoplasm for reactions of the central carbon and iron metabolism of anammox bacteria[6,7].

Central carbon metabolism of anammox bacteria in EET-dependent anammox process

Our analysis showed upregulation under electrode-dependent anammox process of the genes in the Wood-Ljungdahl pathway for CO_2 fixation and acetyl-CoA synthesis (Supplementary Fig. 10, Supplementary Table 6). Also, the key enzyme for CO_2 fixation via the reductive tricarboxylic acid cycle (rTCA) pyruvate:ferredoxin oxidoreductase (PFdO) was upregulated under electrode-dependent anammox process (Supplementary Fig. 10, Supplementary Table 6). This enzyme can catalyze the decarboxylation of pyruvate with use of ferredoxins[34]. Apart from serving as the main electron donor in anammox bacteria, NH_4^+ is also assimilated for biosynthesis via glutamate synthase (GltS). Multiple copies of GltS were found expressed in our analysis (Supplementary Fig. 10, Supplementary Table 3). GltS catalyzes the binding of the ammonium-nitrogen to 2-oxoglutarate with the oxidation of Fd_{red}[35]. The 2-oxoglutarate used for this reaction can be provided by the key enzyme of the rTCA cycle 2-oxoglutarate:ferredoxin oxidoreductase (OGOR)[36]. Multiple copies of OGOR were expressed similarly under both types of electron acceptor (Supplementary Fig. 10, Supplementary Table 3). These enzymes depend on the reducing power of reduced ferredoxin (Fd_{red}) for the reactions, which are the proposed soluble electron carriers in the cytoplasm.

Iron assimilation in anammox bacteria in EET-dependent anammox process

Iron is the fourth most abundant element in Earth's crust[37] and plays an essential role in anammox metabolism. Energy conservation in anammox bacteria depends on iron-containing proteins (i.e.,

cytochromes and iron-sulfur proteins)[38]. Surprisingly the proteins involved in iron transport and assimilation are still unknown. Our analysis revealed that in the absence of soluble electron acceptors (i.e., NO_2^-, NO_3^-), *Ca*. Brocadia electricigens expressed two gene clusters encoding a siderophore-mediated iron uptake system (Supplementary Fig. 10, Supplementary Tables 3 and 13). The expressed siderophore-mediated transport system, which was previously believed to be absent in anammox bacteria[38], is a homolog to the well-studied TonB-dependent Fe(III) uptake complex present in Gram-negative bacteria[39]. Fe(III) uptake relies on beta-barrel TonB-dependent receptors in the outer membrane[40] and an energy-transducing protein complex TonB-ExbB-ExbD that links the outer with the inner membrane and generate a proton motive force[39]. A periplasmic iron-binding protein and an ATP-dependent ABC transporter permease are responsible for the Fe(III)-siderophore translocation across the inner membrane into the cytoplasm, where the Fe(III) is reduced to Fe(II) and released from the complex[39] (Supplementary Fig. 10). Fe(III) reduction in the cytoplasm can be carried out by ferric-chelate reductases/rubredoxins, from which multiple genes were found to be expressed (Supplementary Fig. 10, Supplementary Table 3). After being reduced, the iron can be assimilated into the metalloprosthetic groups of protein complexes[38]. Even though Fe(III) was not added in the experimental setup, *Ca*. Brocadia electricigens may have activated this system in order to uptake Fe(III) as an alternative electron acceptor as well as for iron uptake for assimilation. This finding is in agreement with a previous study using the EET-capable model bacteria *Geobacter sulfurreducens*[41], in which it was shown that the pathways required for EET and metal oxide reduction are distinct.

Supplementary References:

1. Light, S. H. *et al.* A flavin-based extracellular electron transfer mechanism in diverse Gram-

positive bacteria. *Nature* **562**, 140–144 (2018).

2. Hu, Z., Wessels, H. J. C. T., van Alen, T., Jetten, M. S. M. & Kartal, B. Nitric oxide-dependent anaerobic ammonium oxidation. *Nat. Commun.* **10**, 1244 (2019).

3. Ishii, S., Suzuki, S., Tenney, A., Nealson, K. H. & Bretschger, O. Comparative metatranscriptomics reveals extracellular electron transfer pathways conferring microbial adaptivity to surface redox potential changes. *ISME J.* **12**, 2844–2863 (2018).

4. Almeida, N. M. De *et al.* Immunogold Localization of Key Metabolic Enzymes in the Anammoxosome and on the Tubule-Like Structures of *Kuenenia stuttgartiensis*. *J. Bacteriol.* **197**, 2432–2441 (2015).

5. Kartal, B. *et al.* Molecular mechanism of anaerobic ammonium oxidation. *Nature* **479**, 127–130 (2011).

6. Kartal, B. *et al.* How to make a living from anaerobic ammonium oxidation. *FEMS Microbiol. Rev.* **37**, 428–461 (2013).

7. Almeida, N. M. De *et al.* Membrane-bound electron transport systems of an anammox bacterium: A complexome analysis. *Biochim. Biophys. Acta - Bioenerg.* **1857**, 1694–1704 (2016).

8. Oshiki, M., Ali, M., Shinyako-hata, K., Satoh, H. & Okabe, S. Hydroxylamine-dependent Anaerobic Ammonium Oxidation (Anammox) by *Candidatus* Brocadia sinica. *Env. Microbiol* **18**, 3133–3143 (2016).

9. Kostera, J., McGarry, J. & Pacheco, A. A. Enzymatic interconversion of ammonia and nitrite: The right tool for the job. *Biochemistry* **49**, 8546–8553 (2010).

10. He, Z. *et al.* Electricity Production Coupled to Ammonium in a Microbial Fuel Cell. *Environ. Sci. Technol* **43**, 3391–3397 (2009).

11. Qu, B., Fan, B., Zhu, S. & Zheng, Y. Anaerobic ammonium oxidation with an anode as the electron acceptor. *Environ. Microbiol. Rep.* **6**, 100–105 (2014).

12. Vilajeliu-Pons, A. *et al.* Microbial electricity driven anoxic ammonium removal. *Water Res.* **130**, 168–175 (2018).

13. Zhan, G., Li, D., Tao, Y. & Zhu, X. Ammonia as carbon-free substrate for hydrogen production in bioelectrochemical systems. *Int. J. Hydrogen Energy* **39**, 11854–11859 (2014).

14. Zhan, G. *et al.* Autotrophic nitrogen removal from ammonium at low applied voltage in a single-compartment microbial electrolysis cell. *Bioresour. Technol.* **116**, 271–277 (2012).

15. Zhan, G., Zhang, L., Tao, Y., Wang, Y. & Zhu, X. Anodic ammonia oxidation to nitrogen gas catalyzed by mixed biofilms in bioelectrochemical systems. *Electrochim. Acta* **135**, 345–350 (2014).

16. Ruiz-Urigüen, M., Steingart, D. & Jaffé, P. R. Oxidation of ammonium by Feammox *Acidimicrobiaceae* sp. A6 in anaerobic microbial electrolysis cells. *Environ. Sci. Water Res. Technol.* **5**, 1582–1592 (2019).

17. Ruiz-Urigüen, M., Shuai, W. & Jaffé, P. R. Electrode Colonization by the Feammox Bacterium *Acidimicrobiaceae* sp. Strain A6. *Appl. Environ. Microbiol.* **84**, e02029-18 (2018).

18. Kartal, B. & Keltjens, J. T. Anammox Biochemistry: a Tale of Heme c Proteins. *Trends Biochem. Sci.* **41**, 998–1011 (2016).

19. Dietl, A. *et al.* The inner workings of the hydrazine synthase multiprotein complex. *Nature* **527**, 394–397 (2015).

20. Ferousi, C. *et al.* Discovery of a functional, contracted heme-binding motif within a

multiheme cytochrome. *J. Biol. Chem.* **294**, 16953–16965 (2019).

21. Akram, M. *et al.* A nitric oxide-binding heterodimeric cytochrome c complex from the anammox bacterium *Kuenenia stuttgartiensis* binds to hydrazine synthase. *J. Biol. Chem.* **294**, 16712–16728 (2019).

22. Akram, M. *et al.* A 192-heme electron transfer network in the hydrazine dehydrogenase complex. *Sci. Adv.* **5**, eaav4310 (2019).

23. Kumar, A. *et al.* The ins and outs of microorganism–electrode electron transfer reactions. *Nat. Rev. Chem.* **1**, 0024 (2017).

24. Shi, L. *et al.* Extracellular electron transfer mechanisms between microorganisms and minerals. *Nat. Publ. Gr.* **14**, 651–662 (2016).

25. Gescher, J. S., Cordova, C. D. & Spormann, A. M. Dissimilatory iron reduction in *Escherichia coli*: identification of CymA of *Shewanella oneidensis* and NapC of E. coli as ferric reductases. *Mol. Microbiol.* **68**, 706–719 (2008).

26. Beckwith, C. R. *et al.* Characterization of MtoD from *Sideroxydans lithotrophicus*: A cytochrome c electron shuttle used in lithoautotrophic growth. *Front. Microbiol.* **6**, 332 (2015).

27. Luo, S., Guo, W., Nealson, K. H., Feng, X. & He, Z. ^{13}C Pathway Analysis for the Role of Formate in Electricity Generation by *Shewanella Oneidensis* MR-1 Using Lactate in Microbial Fuel Cells. *Sci. Rep.* **6**, 1–8 (2016).

28. Strous, M. *et al.* Deciphering the evolution and metabolism of an anammox bacterium from a community genome. *Nature* **440**, 790–794 (2006).

29. Kane, A. L. *et al.* Formate Metabolism in *Shewanella oneidensis* Generates Proton Motive Force and Prevents Growth without an Electron Acceptor. *J. Bacteriol.* **198**, 1337–1346

(2016).

30. Kracke, F., Vassilev, I. & O. Krömer, J. Microbial electron transport and energy conservation – the foundation for optimizing bioelectrochemical systems. *Front. Microbiol.* **6**, 1–18 (2015).

31. Liu, Y. *et al.* A trans-outer membrane porin-cytochrome protein complex for extracellular electron transfer by *Geobacter sulfurreducens* PCA. *Environ. Microbiol. Rep.* **6**, 776–785 (2014).

32. Shi, L., Fredrickson, J. K. & Zachara, J. M. Genomic analyses of bacterial porin-cytochrome gene clusters. *Front. Microbiol.* **5**, 1–10 (2014).

33. Dantas, J. M. *et al.* Solution structure and dynamics of the outer membrane cytochrome OmcF from *Geobacter sulfurreducens*. *BBA - Bioenerg.* **1858**, 733–741 (2017).

34. Ikeda, T. *et al.* Anabolic five subunit-type pyruvate:ferredoxin oxidoreductase from *Hydrogenobacter thermophilus* TK-6. *Biochem Biophys Res Commun.* **340**, 76–82 (2006).

35. Heuvel, R. H. H. Van Den, Curti, B., Vanoni, M. A. & Mattevi, A. Glutamate synthase: a fascinating pathway from L-glutamine to L-glutamate. *Cell Mol Life Sci.* **61**, 669–681 (2004).

36. Chen, P. Y. *et al.* A Reverse TCA Cycle 2-Oxoacid: Ferredoxin Oxidoreductase that Makes C-C Bonds from CO_2. *Joule* **3**, 595–611 (2018).

37. Weber, K. A., Achenbach, L. A. & Coates, J. D. Microorganisms pumping iron: anaerobic microbial iron oxidation and reduction. *Nat Rev Microbiol.* **4**, 752–764 (2006).

38. Ferousi, C. *et al.* Iron assimilation and utilization in anaerobic ammonium oxidizing bacteria. *Curr. Opin. Chem. Biol.* **37**, 129–136 (2017).

39. Noinaj, N., Guillier, M., Barnard, T. J. & Buchanan, S. K. TonB-Dependent Transporters:

Regulation , Structure , and Function. *Annu Rev Microbiol* **64**, 43–60 (2010).

40. Mosbahi, K., Wojnowska, M., Albalat, A. & Walker, D. Bacterial iron acquisition mediated by outer membrane translocation and cleavage of a host protein. *Proc Natl Acad Sci U S A.* **115**, 6840–6845 (2018).

41. Jiménez Otero, F., Chan, C. H. & Bond, D. R. Identification of Different Putative Outer Membrane Electron Conduits Necessary for Fe(III) Citrate, Fe(III) Oxide, Mn(IV) Oxide, or Electrode Reduction by *Geobacter sulfurreducens*. *J. Bacteriol.* **200**, e00347-18 (2018).

Supplementary figures

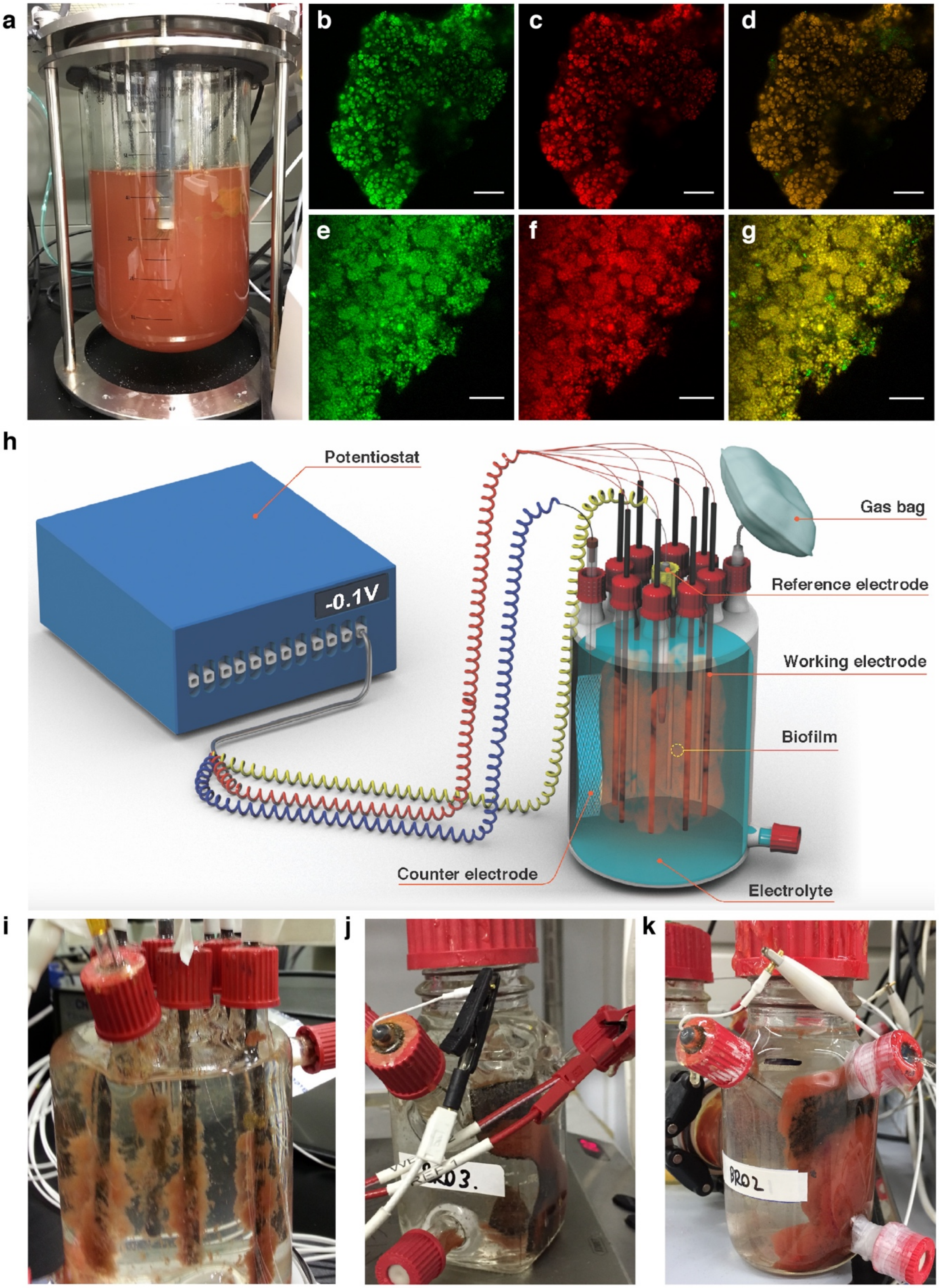

Supplementary Fig. 1 Reactors used in this study. (**a**) Photograph of membrane bioreactor (MBR) used for the enrichment of anammox planktonic cells. (**b**-**g**) Confocal laser scanning microscopy images of enriched biomass of *Ca*. Brocadia (**b**-**d**) and *Ca*. Scalindua (**e**-**g**). The images are showing all bacteria (green), anammox bacteria (red) and the merged micrograph (yellow). Fluorescence *in-situ* hybridization was performed with EUB I, II and III probes for all bacteria and Alexa647-labeled Amx820 probe for anammox bacteria. The scale bars represent 20 μm in length. (**h**) Schematic representation of the multiple working electrode microbial electrolysis cell (MEC). Photographs of the single-chamber multiple working electrode MEC (**i**); single-chamber MEC with a single working electrode (**j**) and double-chamber MEC with a single working electrode (**k**) with anammox biofilm shown on all the electrodes.

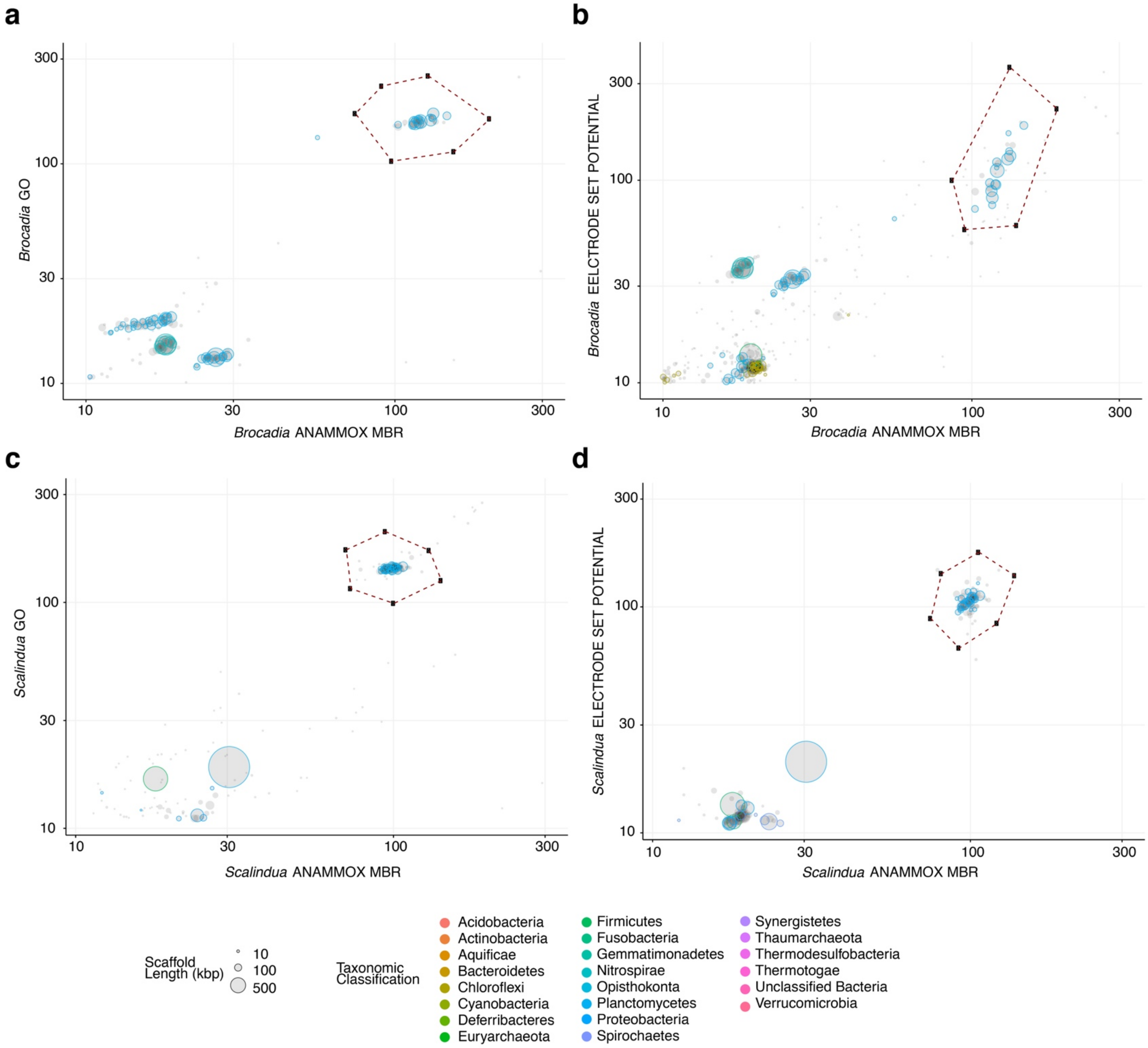

Supplementary Fig. 2 Metagenome scaffolds from graphene oxide and microbial electrolysis cells. (**a-d**) Differential coverage binning of the genome sequences from the incubation of *Ca*. Brocadia and *Ca*. Scalindua with graphene oxide (GO) (**a** and **c**) or working electrode (0.6 V vs. standard hydrogen electrode (SHE) applied potential) as the sole electron acceptor (**b** and **d**). Each

circle represents a metagenomic scaffold, with size proportional to scaffold length; only scaffolds ≥ 5 Kbp are shown. Taxonomic classification is indicated by color; clusters of similarly colored circles represent potential genome bins. The x and y-axes show the sequencing coverage in the samples (log-scaled). Extracted anammox genomes are enclosed by dashed polygons.

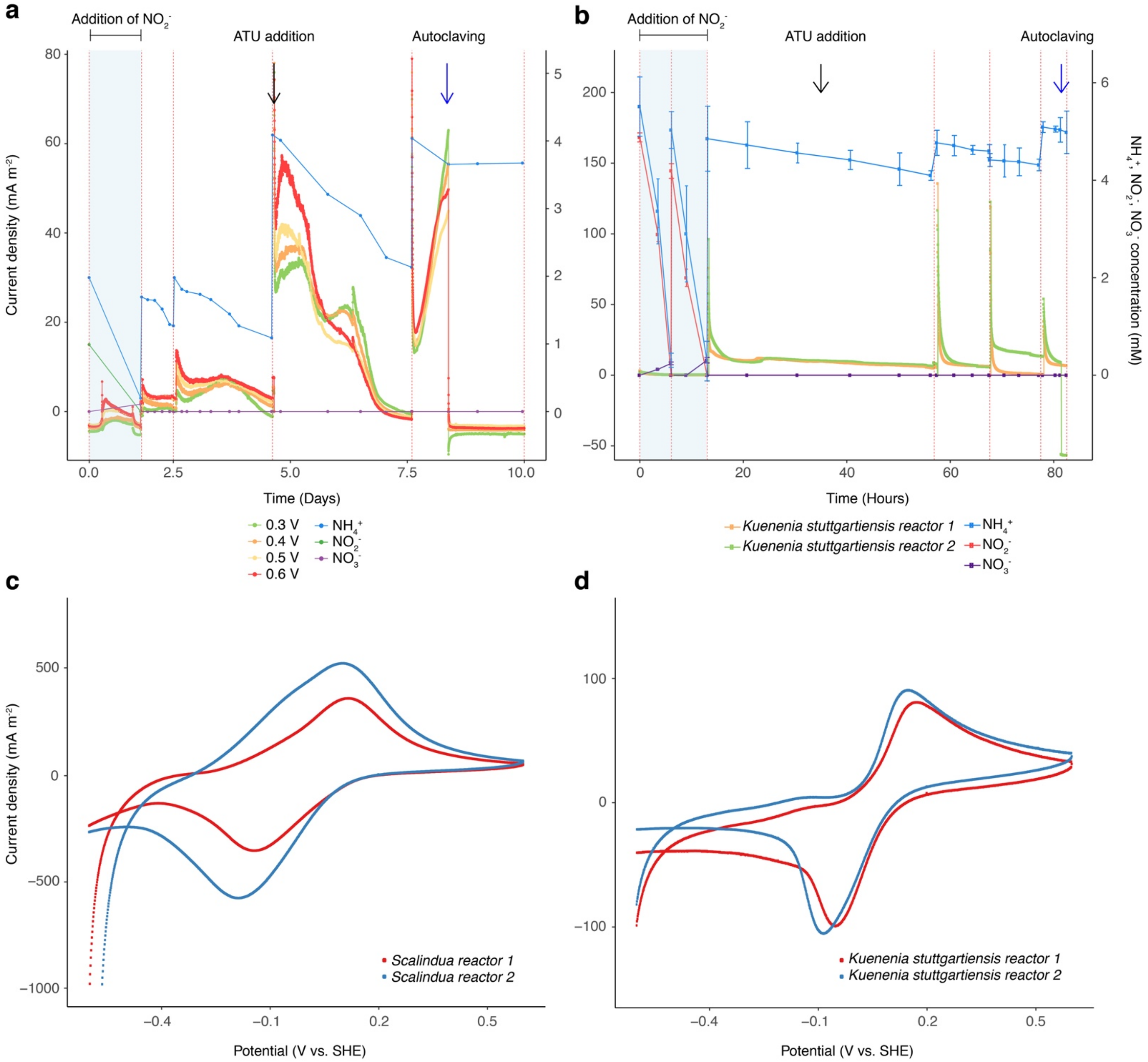

Supplementary Fig. 3 *Ca.* Scalindua and *Kuenenia stuttgartiensis* are electrochemically active. (**a** and **b**) Ammonium oxidation coupled to current generation in chronoamperometry experiment conducted in (**a**) one single-chamber multiple working electrode microbial electrolysis cell (MEC) inoculated with *Ca*. Scalindua and operated under different set potentials and (**b**)

duplicate single-chamber MECs inoculated with *K. stuttgartiensis* and operated with a working electrode at 0.6 V vs standard hydrogen electrode (SHE). Red dashed lines represent a change of batch. The highlighted area with blue refers to the operation of MEC in the presence of nitrite, which is the preferred electron acceptor for anammox bacteria. The black arrow indicates the addition of allylthiourea (ATU), a compound that selectively inhibits nitrifiers. The black arrow in plot (**b**) indicates ATU addition in reactor 2 of *K. stuttgartiensis*. The blue arrow indicates autoclaving followed by re-connecting of the MECs. The blue arrow in plot (**b**) indicates autoclaving of reactor 2 of *K. stuttgartiensis*. NH_4^+ concentration from duplicate MEC reactors in plot (**b**) are presented as mean ± SD. (**c** and **d**) Cyclic Voltammogram (1 mV s^{-1}) of *Ca*. Scalindua (**c**) and *K. stuttgartiensis* (**d**) biofilm grown on the anode.

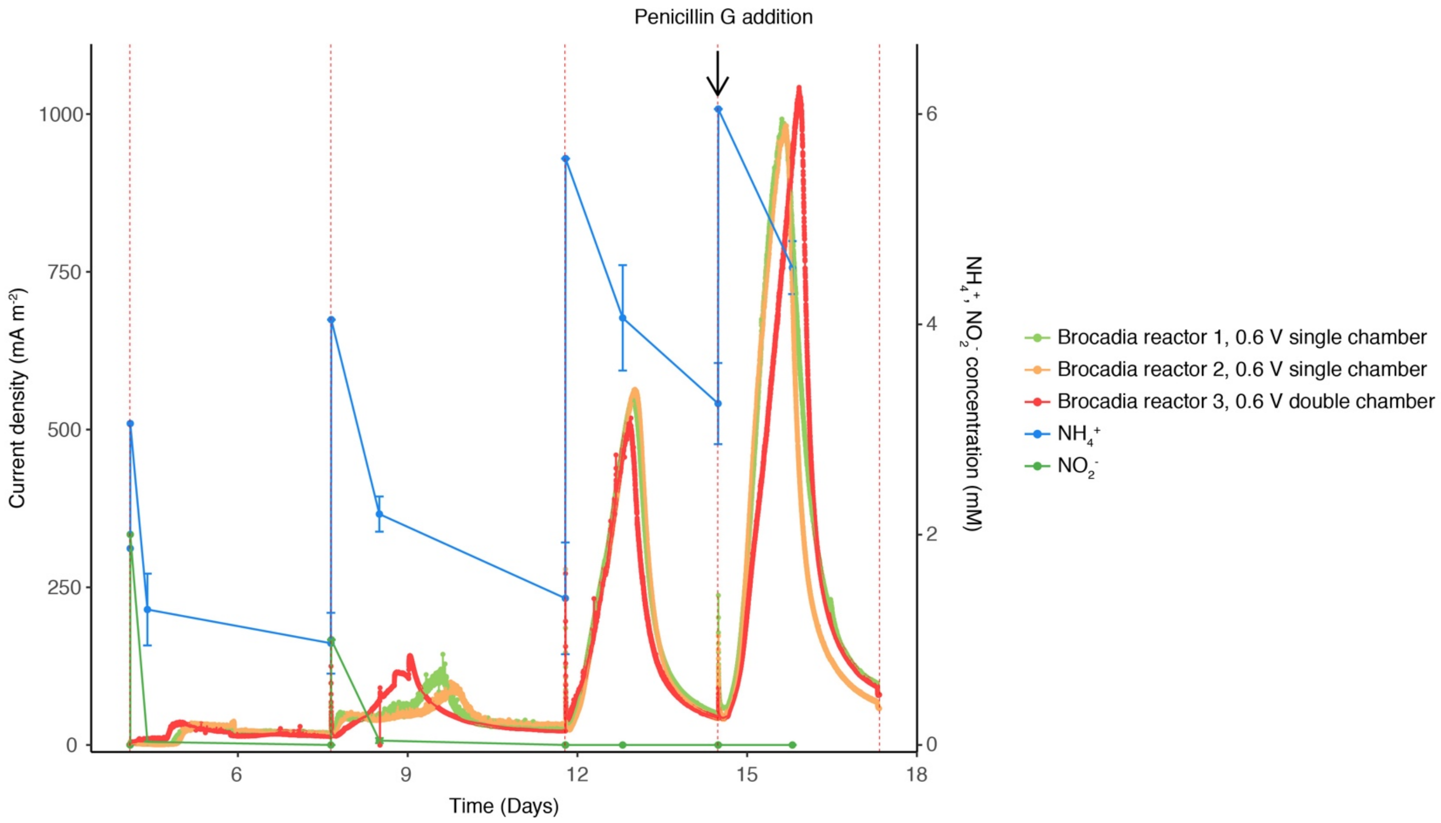

Supplementary Fig. 4 Influence of cathodic reaction (i.e., hydrogen evolution reaction). Ammonium oxidation and chronoamperometry of single and double-chamber microbial electrolysis cells (MECs) inoculated with *Ca*. Brocadia and operated at a set potential of 0.6 V vs. standard hydrogen electrode (SHE). Red dashed lines represent a change of batch. The black arrow indicates the addition of penicillin G to reactor 2. Penicillin G is not active against anammox bacteria but inhibits the activity of most heterotrophs. NH_4^+ concentration from triplicate MEC reactors are presented as mean ± SD.

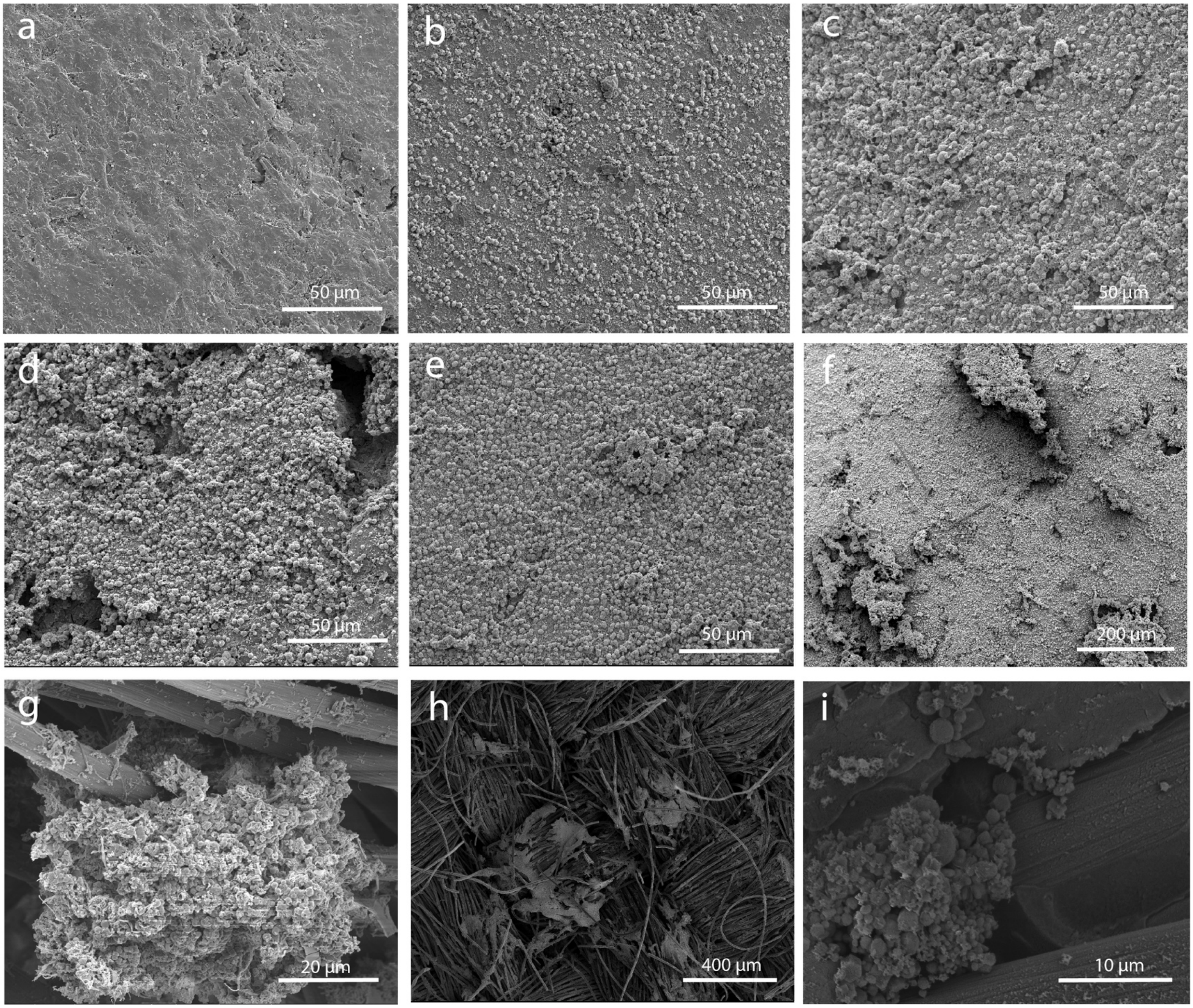

Supplementary Fig. 5 Micrographs of anammox biofilm on working electrodes. (**a-f**) Scanning electron microscopy (SEM) images of *Ca*. Brocadia biofilm grown on graphite rod anodes after 55 days of operation at set potential of 0.2 V (**a**), 0.3 V (**b**), 0.4 V (**c**), 0.5 V (**d**) and 0.6 V (**e** and **f**) vs. standard hydrogen electrode (SHE). (**g**) SEM image showing *Ca*. Scalindua

biofilm grown on carbon cloth anode at a set potential of 0.6 V vs. SHE. (**h** and **i**) SEM images of *Kuenenia stuttgartiensis* biofilm grown on carbon cloth anode at 0.6 V vs. SHE.

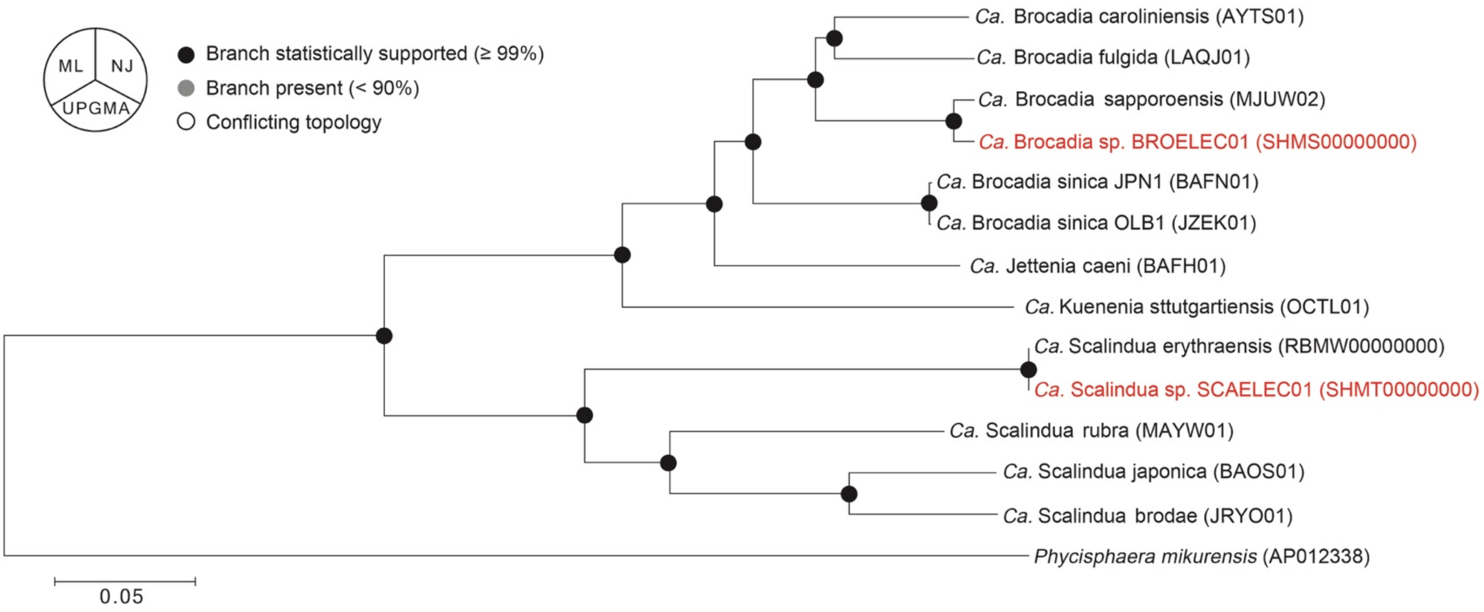

Supplementary Fig. 6 Phylogenomics analysis of anammox genomes extracted from electrodes. Pie charts at the nodes represent the bootstrap support values and the bootstrap consensus inferred from 1000 iterations. Support value ≥ 99% is filled with black. ML, maximum likelihood method; NJ, neighbor joining method; and UPGMA, unweighted pair group method with arithmetic mean. The anammox genomes extracted from the biofilm community on the working electrodes are shown in red. GenBank accession numbers for each genome are provided in parentheses. Sequences of two different strains of *Brocadia sinica* were used as a reference of same species genomes. The sequence of a member from the phylum planctomycetes different than anammox bacteria was used as outgroup.

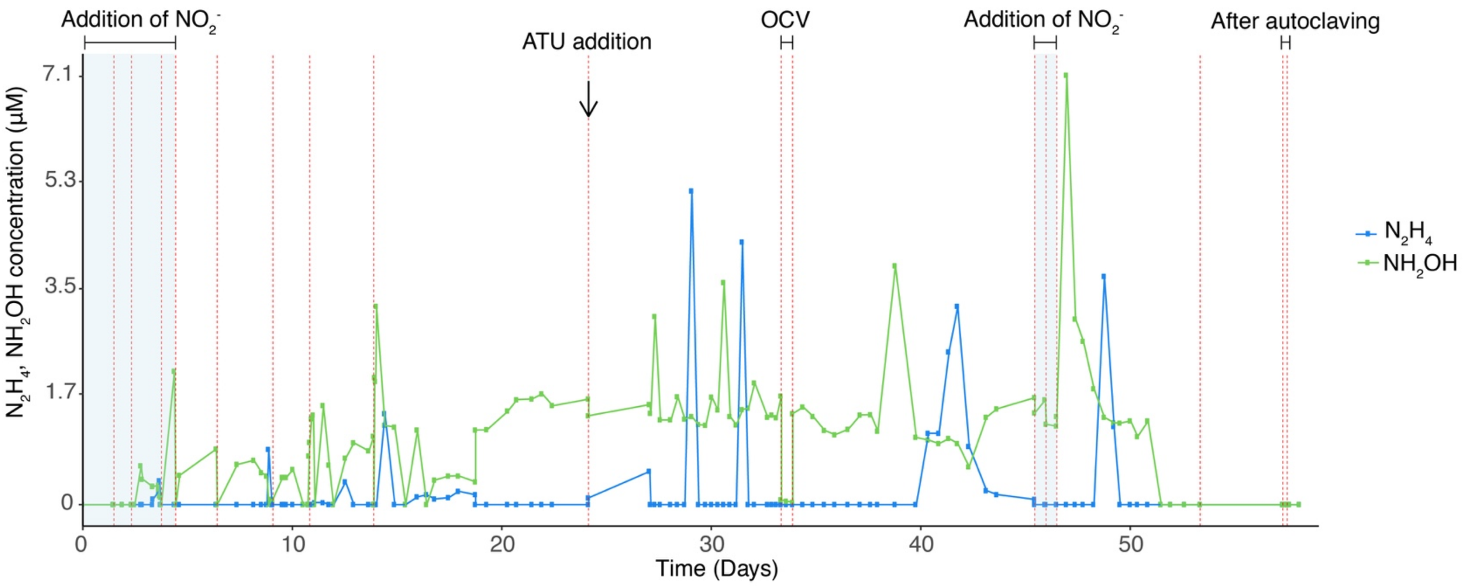

Supplementary Fig. 7 NH_2OH and N_2H_4 concentration in single-chamber multiple working electrode microbial electrolysis cell inoculated with *Ca.* Brocadia. Red dashed lines represent a change of batch. The highlighted area in blue refers to the operation of **microbial electrolysis cell (**MEC) in the presence of nitrite, which is the preferred electron acceptor for anammox bacteria. The black arrow indicates the addition of allylthiourea (ATU). OCV indicates MEC operated under open circuit voltage.

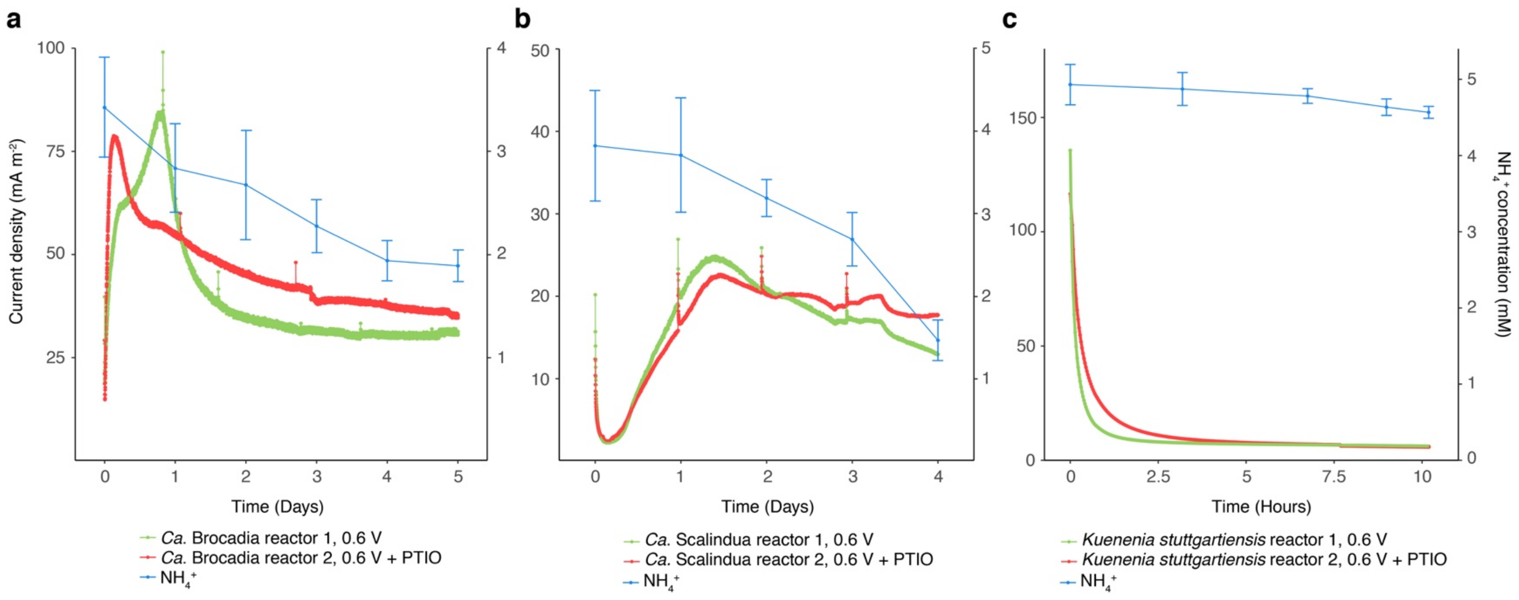

Supplementary Fig. 8 Influence of PTIO (a NO-scavenger). (**a**) Ammonium oxidation and chronoamperometry of *Ca*. Brocadia single-chamber duplicate MECs with and without PTIO addition. (**b**) Ammonium oxidation and chronoamperometry of *Ca*. Scalindua single-chamber MECs with and without PTIO addition. (**c**) Ammonium oxidation and chronoamperometry of *Kuenenia stuttgartiensis* single-chamber MECs with and without PTIO addition. NH_4^+ concentration from duplicate MEC reactors in plot are presented as mean ± SD.

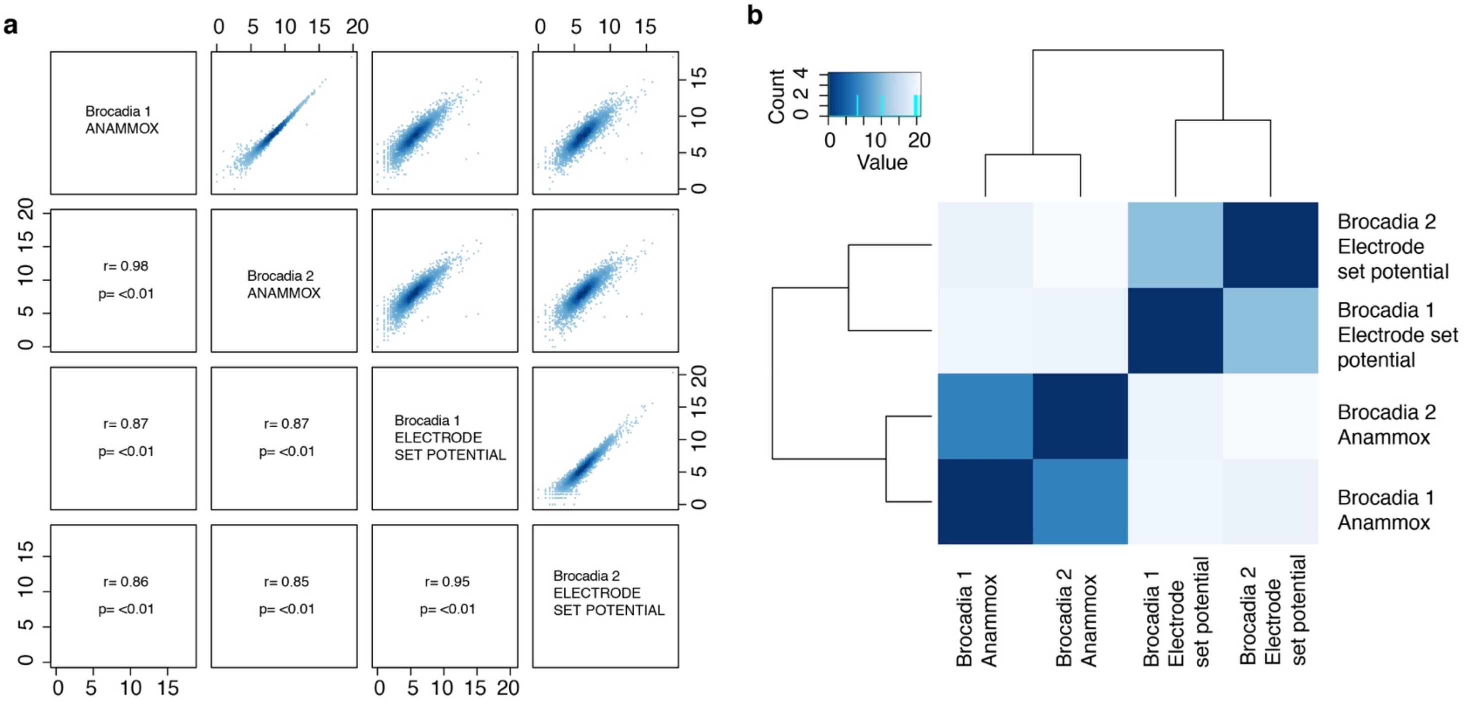

Supplementary Fig. 9 Transcriptomics similarity between biological replicate samples. (a) Pairwise overview of *Brocadia* transcriptomics samples. "Brocadia Anammox" corresponds to the experimental condition where nitrite was used as the sole electron acceptor. "Brocadia Electrode set potential" corresponds to the experimental condition where the working electrode (0.6 V vs. SHE) was used as the sole electron acceptor. All counts were normalized to Log2+1 values. The upper right panel shows the normalized counts while the lower-left panel shows the corresponding Pearson correlation coefficient between samples and the P-value. The intra-replicate correlation is high and conversely the inter-replicate correlation is low. This indicates a high similarity between the biological replicates and differentially expressed genes across the experimental setups. (**b**) Heatmap clustering of the sample-to-sample distances. Hierarchical clustering shows that anammox (nitrite as the electron acceptor) and electrode-dependent anammox (set potential of 0.6 V vs. SHE) samples were clearly separated and clustered as independent groups.

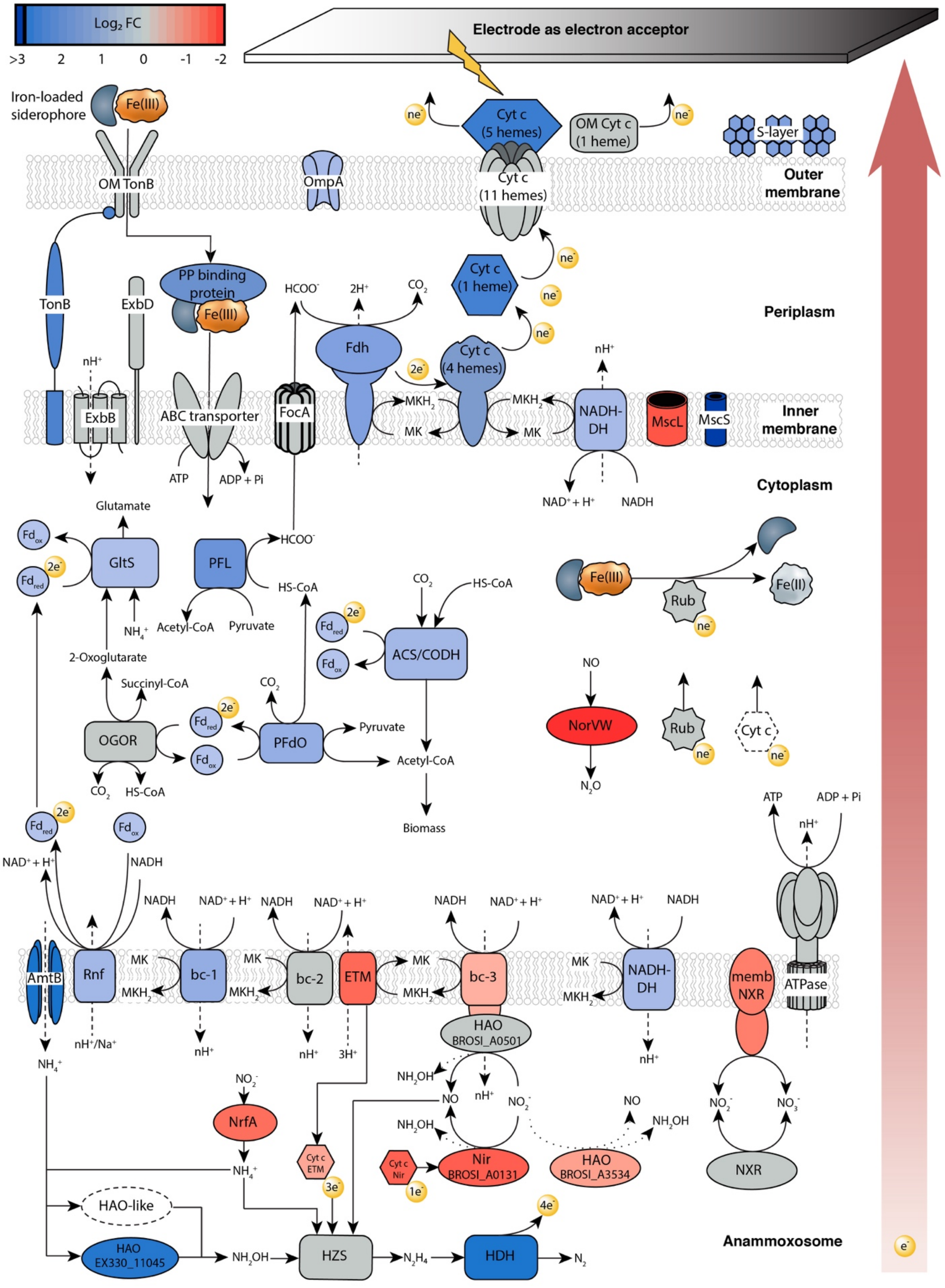

Log₂ FC
>3 2 1 0 -1 -2
Electrode as electron acceptor
Iron-loaded siderophore
Fe(III)
OM TonB
OmpA
Cyt c (5 hemes)
OM Cyt c (1 heme)
Cyt c (11 hemes)
S-layer
Outer membrane
TonB
ExbD
PP binding protein
Cyt c (1 heme)
Periplasm
HCOO⁻
2H⁺
CO₂
Fdh
Cyt c (4 hemes)
nH⁺
ExbB
ABC transporter
FocA
MKH₂
MK
NADH-DH
MscL
MscS
Inner membrane
ATP
ADP + Pi
NAD⁺ + H⁺
NADH
Cytoplasm
Glutamate
GltS
PFL
HS-CoA
Acetyl-CoA
Pyruvate
NH₄⁺
2-Oxoglutarate
Succinyl-CoA
ACS/CODH
Fe(II)
Rub
NO
NorVW
N₂O
Cyt c
OGOR
PFdO
Biomass
AmtB
Rnf
bc-1
bc-2
ETM
bc-3
memb NXR
ATPase
HAO BROSI_A0501
nH⁺/Na⁺
3H⁺
NH₂OH
NO₂⁻
NO₃⁻
NrfA
Cyt c ETM
Cyt c Nir
Nir BROSI_A0131
HAO BROSI_A3534
NXR
HAO-like
HAO EX330_11045
HZS
N₂H₄
HDH
N₂
Anammoxosome

Supplementary Fig. 10 Molecular model of electrode-dependent anaerobic ammonium oxidation. The putative EET metabolic pathway of *Ca.* Brocadia to deliver electrons to an electrode was constructed with the transcriptional changes of selected marker genes in response to the electrode as the electron acceptor. Samples for comparative transcriptomic analysis were taken from mature electrode's biofilm of single-chamber MECs with NO_2^- as the sole electron acceptor and after switching to set potential growth (0.6 V vs. SHE, electrode as the electron acceptor). Log_2 fold changes (Log_2 FC) in the expression are shown as follows; gene and gene clusters are shown in blue if upregulated or red if downregulated relative to the electrode as the electron acceptor. The color grey corresponds to genes that were expressed under similar levels in both conditions (i.e., electrode or nitrite as the electron acceptor). Dashed lines represent proton (H^+) transport across membranes. Dashed curves indicate proteins, reactions or processes that have not been established yet. Genomic identifiers of the proteins used in the model are listed in Supplementary Table 5. The reactions are described in the Supplementary discussion of the paper and are catalyzed by the enzymes ABC transporter: Iron ABC transporter permease and ATP-binding protein; ACS/CODH: Acetyl-CoA synthase/ CO dehydrogenase; AmtB: Ammonium transport protein; ATPase: ATP synthase; bc-1: Rieske/cytochrome b complex; bc-2: Rieske/cytochrome b complex; bc-3: Rieske/cytochrome b complex; Cyt *c* (1 heme): Periplasmic mono-heme *c*-type cytochrome; Cyt *c* (11 hemes): Membrane-anchored undeca-heme cytochrome *c*; Cyt *c* (4 hemes): Membrane-anchored tetraheme *c*-type cytochrome; Cyt *c* (5 hemes): Outer membrane penta-heme *c*-type cytochrome; Cyt *c* ETM: Cytochrome c redox partner of the ETM; Cyt Nir: Cytochrome *c*; ETM: electron transfer module for hydrazine synthesis; ExbB: Biopolymer transport protein ExbB/TolQ; ExbD: Biopolymer transport protein ExbD/TolR; FDH: membrane-bound formate dehydrogenase; Fd_{ox}: Ferredoxin (oxidized); Fd_{red} Ferredoxin

(reduced); FocA: Formate/nitrite transporter; GltS: Glutamate synthase; HAO BROSI_A0501: Hydroxylamine oxidoreductase; HAO BROSI_A3534: Hydroxylamine oxidoreductase; HAO EX330_11045: Hydroxylamine oxidoreductase; HDH: hydrazine dehydrogenase; HZS: hydrazine synthase; membNXR: membrane-bound complex of the *nxr* gene cluster; MscL: Large mechanosensitive channel; MscS: Pore-forming small mechanosensitive channel; NADH-DH: NADH dehydrogenase; Nir BROSI_A0131: nitrite reductase; NorVW: Flavodoxin nitric oxide reductase; NrfA: ammonium-forming nitrite reductase; NXR: nitrite:nitrate oxidoreductase; OGOR: 2-oxoglutarate ferredoxin oxidoreductase; OM Cyt *c* (1 heme): Outer membrane lipoprotein mono-heme c-type cytochrome; OM TonB: TonB-dependent receptor; OmpA: OmpA-like outer membrane protein, porin; PFdO: Pyruvate ferredoxin oxidoreductase; PFL: Pyruvate formate lyase; PP binding protein: Iron ABC transporter periplasmic substrate-binding protein; Rnf: RnfABCDGE type electron transport complex; Rub: Rubredoxin/ferric-chelate reductase; S-layer: S-layer protein; TonB: Energy transducer TonB.
