# Supplementary material for: Extracellular electron transfer-dependent anaerobic oxidation of ammonium by anammox bacteria
Source: Nat Commun. 2020 Apr 28;11:2058. doi: 10.1038/s41467-020-16016-y (PMC7188810; doi:10.1038/s41467-020-16016-y)
Supplement: Supplementary file 4 — Reporting summary [file 41467_2020_16016_MOESM4_ESM.pdf]

nature research

Corresponding author(s): Pascal Saikaly

Last updated by author(s): Mar 5, 2020

Reporting Summary

Nature Research wishes to improve the reproducibility of the work that we publish. This form provides structure for consistency and transparency in reporting. For further information on Nature Research policies, see Authors & Referees and the Editorial Policy Checklist.

Statistics

For all statistical analyses, confirm that the following items are present in the figure legend, table legend, main text, or Methods section.

n/a	Confirmed	
☐	☒	The exact sample size (*n*) for each experimental group/condition, given as a discrete number and unit of measurement
☐	☒	A statement on whether measurements were taken from distinct samples or whether the same sample was measured repeatedly
☒	☐	The statistical test(s) used AND whether they are one- or two-sided<br>*Only common tests should be described solely by name; describe more complex techniques in the Methods section.*
☒	☐	A description of all covariates tested
☒	☐	A description of any assumptions or corrections, such as tests of normality and adjustment for multiple comparisons
☐	☒	A full description of the statistical parameters including central tendency (e.g. means) or other basic estimates (e.g. regression coefficient) AND variation (e.g. standard deviation) or associated estimates of uncertainty (e.g. confidence intervals)
☐	☒	For null hypothesis testing, the test statistic (e.g. *F*, *t*, *r*) with confidence intervals, effect sizes, degrees of freedom and *P* value noted<br>*Give P values as exact values whenever suitable.*
☒	☐	For Bayesian analysis, information on the choice of priors and Markov chain Monte Carlo settings
☒	☐	For hierarchical and complex designs, identification of the appropriate level for tests and full reporting of outcomes
☒	☐	Estimates of effect sizes (e.g. Cohen's *d*, Pearson's *r*), indicating how they were calculated

Our web collection on statistics for biologists contains articles on many of the points above.

Software and code

Policy information about availability of computer code

Data collection	Data from bioelectrochemical analyses was collected with the EC-lab V 10.02 software® of the VMP3 potentiostat (BioLogic Science Instruments, USA). Data from confocal laser scanning microscope Leica SP7 was collected with LAS X Life Science software. GC/MS data was collected with the software Enhanced ChemStation MSD ChemStation E.02.021431
Data analysis	The following software were used for Data analysis: R v. 3.3.4 using the R-studio environment Version 1.0.153, MSD ChemStation E.02.021431, EC-lab V 10.02 LAS X Life Science, DAIME v 2.1, ImageJ V 2.0.0, Cutadapt package v. 1.10, SPAdes v. 3.7.1, minimap2 (v. 2.5), samtools, Prodigal V 2.6, HMMER3 (http://hmmer.janelia.org/), MEGAN6, BLAST (v. 2.2.28+), using SINA (v. 1.2.11), mmgenome package (v. 0.7.1.), CheckM 1.0.10, PROKKA (v. 1.12-beta), anvi'o v 5.4, MEGA7, USEARCH v10.0.2132, BBDuk, minimap2 v2.8-r672, DESeq2 1.18, SignalP 5.0, TMHMM 2.0, PSORTb 3.0.2, HHpred. Workflows describing custom analysis code used in this study are available as described in the Data Availability Statement.

For manuscripts utilizing custom algorithms or software that are central to the research but not yet described in published literature, software must be made available to editors/reviewers. We strongly encourage code deposition in a community repository (e.g. GitHub). See the Nature Research guidelines for submitting code & software for further information.

Data

Policy information about availability of data

All manuscripts must include a data availability statement. This statement should provide the following information, where applicable:

- Accession codes, unique identifiers, or web links for publicly available datasets
- A list of figures that have associated raw data
- A description of any restrictions on data availability

Raw sequencing reads of Illumina HiSeq of metagenomics and metatranscriptomics data generated in this study have been deposited in the NCBI under BioProject PRJNA517785. Annotated GenBank files for the anammox genomes extracted in this study can be found under the accession numbers SHMS00000000 and SHMT00000000. Data for genome binning and comparative transcriptomics analysis are available and entirely reproducible using the R files available on https://

github.com/DarioRShaw/Electro-anammox. Also, complete Datasets generated in the differential expression analysis are available in the Supplementary material. Additional required datasets generated in the current study are available upon request.

Field-specific reporting

Please select the one below that is the best fit for your research. If you are not sure, read the appropriate sections before making your selection.

☒ Life sciences ☐ Behavioural & social sciences ☐ Ecological, evolutionary & environmental sciences

For a reference copy of the document with all sections, see nature.com/documents/nr-reporting-summary-flat.pdf

Life sciences study design

All studies must disclose on these points even when the disclosure is negative.

Sample size	No statistical methods were used to predetermine the sample size. In all experiments, three biological replicates were used, unless mentioned otherwise. No explicit power analysis were carried out.
Data exclusions	No data were excluded from the analysis besides sequencing reads with low quality scores, sequencing reads that did not map to genes within our dataset, and mapped transcripts with extremely low abundance, as is commonly performed in metatranscriptomics analyses.
Replication	All experiments were independently repeated and all attempts to replicate the experiments were successful.
Randomization	The experiments were not randomized, since all analyses concerned to specific enrichment cultures.
Blinding	Investigators were not blinded to group allocation during data collection or analysis.

Reporting for specific materials, systems and methods

We require information from authors about some types of materials, experimental systems and methods used in many studies. Here, indicate whether each material, system or method listed is relevant to your study. If you are not sure if a list item applies to your research, read the appropriate section before selecting a response.

Materials & experimental systems

n/a	Involved in the study	
☒	☐	Antibodies
☒	☐	Eukaryotic cell lines
☒	☐	Palaeontology
☒	☐	Animals and other organisms
☒	☐	Human research participants
☒	☐	Clinical data

Methods

n/a	Involved in the study	
☒	☐	ChIP-seq
☒	☐	Flow cytometry
☒	☐	MRI-based neuroimaging
